# The Dental-BIOfilm Detection TECHnique (D-BioTECH): A Proof of Concept of a Patient-Based Oral Hygiene

**DOI:** 10.3390/medicina58040537

**Published:** 2022-04-13

**Authors:** Roberta Grassi, Gianna Maria Nardi, Marta Mazur, Roberto Di Giorgio, Livia Ottolenghi, Fabrizio Guerra

**Affiliations:** 1 Department of Biomedical Sciences, University of Sassari, 07100 Sassari, Italy; 2 Department of Dental and Maxillofacial Sciences, Sapienza University of Rome, 00161 Rome, Italy; giannamaria.nardi@uniroma1.it (G.M.N.); marta.mazur@uniroma1.it (M.M.); roberto.digiorgio@uniroma1.it (R.D.G.); livia.ottolenghi@uniroma1.it (L.O.); fabrizio.guerra@uniroma1.it (F.G.)

**Keywords:** dentistry, oral hygiene, oral rehabilitation

## Abstract

To date, no strong long-term data have been reported about new innovative clinical protocols to manage oral hygiene. An improper management of oral hygiene may lead to an increase in dental implant failure, and to an increase in infective complications in prosthetic rehabilitation. Personalized techniques are strongly required in dentistry and dental hygiene. A customized and personalized approach to oral hygiene is crucial in ensuring not only effective treatment, but also a careful analysis of the general health status of the patient involved in the therapeutic process. D-BioTECH is an acronym for Dental BIOfilm Detection Technique: it is based on a tailored approach to patients, ensuring that the operator actively interacts with the patient and their specific needs, especially during the domiciliary therapy. D-BioTECH is an approach to preventive care: in D-BioTECH, both dental hygienists and dentists play a central role. The use of a personalized approach to oral hygiene is the first step towards increasing implant and prosthesis survival rate; moreover, personalized medicine is strategic for managing and preventing the biological complications associated with several dental risk factors.

## 1. Introduction

Current etiopathogenetic models define the pathologies of the periodontium as deriving from altered homeostasis at a tissue support level—the action of a modified microbiota can lead to inflammation and progressive tissue damage. Periodontitis is an inflammatory condition starting from bacteria-rich dental plaque. Periodontal inflammation starts from microbial dysbiosis, but it should be considered a multifactorial disease, where genetic factors, immune response, and proper oral hygiene may contribute to the onset and severity of the disease [1]. Some bacterial species are able to modulate the immune response, favoring the change from a symbiotic to a dysbiotic population. *Porphyromonas gingivalis* is a key species in this sense. Specific prostheses for lysine and cysteine (Rgp and Kgp, respectively), called the gingipain complex, are important virulence factors involved in this mechanism, as they are able to cleave IgG1, and interfere with the complement system and with some cytokines [2].

Peri-implantitis represents the disease condition equivalent to the periodontal one in the context of peri-implant tissues. This does not mean that the etiopathological model represents the perfect replica: the clinic suggests, for example, that peri-implant damage tends to be more extensive and progressive. Differences have also been identified at the microbiological level: for example, some studies have found higher concentrations of *Tannerella forsythia* in peri-implant lesions (compared to periodontal pockets), suggesting that the pathogenetic significance of the bacterium may also be higher [3,4].

Several bacteria in the oral cavity are associated with the onset of periodontitis: the most recognized are typically *Aggregatibacter actinomycetemcomitans*, *Porphyromonas gingivalis*, *Prevotella intermediate*, *Tannerella forsythia*, *Treponema denticola*, *Prevotella nigrescens*, *Capnocytophaga sputigena*, *Eikenella corrodens*, Fusobacterium nucleatum, *Campylobacter rectus* [5]. These bacteria can act against cells of the gingival epithelium, binding the β-1 integrins. Furthermore, the Toll-like receptors (TLRs) located on the cell membrane of the gingival epithelium can recognize the lipoproteins, called Pathogen-associated molecular patterns (PAMPs), and several transcription factors, such as Nuclear Factor-kB (NF-kB) and Activator Protein-1 (AP-1), which together trigger the inflammatory chain. The activation of cytokines and chemokines is a stimulus that attracts leukocytes in the periodontal space [6,7].

The periodontal inflammation can be stopped within its first weeks if oral hygiene is properly managed. D-BioTECH is an acronym for the Dental BIOfilm Detection Technique: it is based on an approach, tailored to patients, that ensures that the operator actively interacts with the patient and their specific needs, especially during domiciliary therapy. This approach is likely to prevent the irreversible processes that lead to chronic periodontitis [8].

### 1.1. Incidence of Periodontal Diseases in the General Population and Main Pathogenic Processes

Periodontal diseases are highly diffuse in the general population. The recent Global Burden of Disease Study (GBD, 1990–2019) indicated that, in 2019, there were 1.1 B (95% uncertainty interval: 0.8–1.4 billion) prevalent cases of severe periodontitis globally. From 1990 to 2019, the age-standardized prevalence rate of severe periodontitis increased by 8.44% (6.62–10.59%) worldwide [9]. According to data published by the Italian Society of Periodontology in 2015, 60 to 70% of adults in Italy are affected by periodontal disease; furthermore, the incidence is most dramatic in adults in the range of 35–44 years old [10].

Low-income countries have a higher prevalence of plaque accumulation among children. On the other hand, Europe and North America have a prevalence of gingivitis ranging from 70 to 95% in adults. Similarly, the percentage of adolescents with high levels of bacterial biofilm ranges from 35% to 70% in developing countries, and from 4% to 34% in developed countries [11]. Several risk factors, such as smoking, diabetes, drugs, and so on, can impact the incidence of periodontal diseases; however, the factor with the most impact is poor oral hygiene [12].

According to the most recent epidemiological studies, the distribution of periodontitis in women and in men is different: severe periodontitis is more frequent in men compared to women. A possible explanation for this statistic is that there are sex-related differences in immune response to bacterial infection [13,14].

To date, periodontitis affects about 60% of the adult population, and the incidence of periodontal diseases tends to increase over time [15,16].

Periodontitis affects about 30% of subjects aged between 25 and 29, and over 50% of subjects between the ages of 55 and 64 years [17].

The effects of periodontitis are quite different in adults and adolescents. In adolescents, periodontal disease occurs as a result of poor oral hygiene with a predominant genetic component [18]. Typically, bacterial infection leads to gum inflammation and the consequent development of periodontal pockets [19]. The first form of periodontitis that occurs in adolescents is gingivitis affecting between 40% and 60% of school-age children in the United States; however, severe periodontitis is less frequent, affecting less than 1% of North Americans and up to 3% of Africans [20].

The diagnosis of periodontal pathology in children is complex: it is important to make a diagnosis before bone loss in the primary dentition, as this may lead periodontal damage that will permanently impact tooth vitality [21,22].

On the contrary, adults can develop morphological changes to their dental roots that lead to a predisposition to gum recession; hence, this begins the onset of generalized periodontitis. In the elderly, bacterial infection sustained by poor oral hygiene leads to bone recession; periodontal diseases are highly worsened by mobile and fixed prostheses [23,24].

### 1.2. Current Methods to Detect Dental Plaque

The detection of dental plaque is an important procedure, and often strategic in oral hygiene management. A proper detection of bacterial plaque is helpful to improve the effectiveness of mouth disinfection, and it can drastically reduce the onset of periodontal diseases. The revelation of dental plaque is often challenging; it may be performed either by clinical observation of the tooth surface—e.g., searching for colour changes—or by analysing the variation of natural tooth fluoresce under blue light. On the market there are various types of plaque detectors that have different approaches to this clinical issue. Currently, practitioners mostly use fluorescein to detect dental plaque. This fluorescent substance has the particularity of being colourless in normal light; however, under specific light, fluorescein reacts, highlighting the area that is strongly linked to plaque [25].

Several studies have been conducted on the reliability of fluorescein as a technique for detecting dental plaque and caries. In particular, the red fluorescence of dental plaque originating from the porphyrins in oral bacteria can allow the visualization, detection, and score of plaque without revealing agents [26].

According to some recent studies, the detection of dental plaque can also be properly performed with alternative methods. The classical fluorescence is currently used and well documented [27,28]. An Italian team of researchers validated the efficiency of the matrix metalloproteinase-8 (MMP-8) chair-side test; the presence of MMP-8 in the oral cavity correlates with the progression and severity of periodontitis-related clinical parameters, i.e., an increase in gingival bleeding, loss of gingival attachment, and increased depth of the periodontal pockets [27,28].

### 1.3. Current Methods to Remove Dental Plaque

Numerous pieces of research conducted over the years have largely demonstrated that plaque control can help to prevent the onset of periodontal diseases. Studies conducted in vivo on animal models have shown that, in the absence of plaque, the development of gingivitis cannot be induced, especially with regard to periodontitis. Consequentially, clinical tests conducted in vivo on humans have shown that the presence of plaque is synonymous with the onset of diseases of the periodontium. These studies have also shown that, the complete resolution of the inflammatory process was followed by the removal of plaque through proper home-based oral hygiene procedures [29].

The removal of dental caries is treated by invasive techniques aimed at removing contaminated dental tissue; however, different methods offer new approaches, such as laser therapy [30].

Normal plaque removal is performed through mechanical and chemical procedures. Mechanical procedures are carried out with periodontal curettes and ultrasonic scalers, while chemical procedures are carried out with citric acid, H2O2, and chlorhexidine digluconate, combined with the use of local or systemic antibiotics to reduce the inflammatory state [31].

As opposed to traditional manual instruments, nowadays more modern techniques are preferred. These make use of ultrasonic instruments that, for example, use the high-frequency oscillation of a blunt metal tip for breaking and removing deposits [30,31,32,33]. Ultrasound debridement is based on different mechanisms, i.e., mechanical disruption, irrigation at high pressure, cavitation, and acoustic microstreaming. Mechanical movements are at the core of the vibratory action of the oscillating metal tip working against the dental plaque. Irrigation supports the mechanical action of water flowing from the tip, so that high-pressure washing can remove plaque and food deposits from the surface of the teeth. Cavitation allows to break up and removal of dental biofilm; finally, microstreaming induces a reduction in the biofilm from the turbulent water currents around the working tip [32]. Recently, a novel technique has been developed in order to achieve a simple and safe removal of oral biofilm; this new technique is based on the action of fine water droplets sprayed at high speed onto the tooth’s surface. The advantage of this technology is the ability to remove plaque from covered and poorly accessible surfaces to avoid potential infection at sites not properly treated. In detail, the water droplets have a diameter of about 40μm and a very low mass, despite their high kinetic energy [33]. Modern literature also reports new innovative therapeutic approaches; for example, a laser coupled with photodynamic therapy (PDT) used in implant sites has been demonstrated to be effective [34].

In this context, laser therapy may be an option for the treatment of periodontally compromised patients. The use of non-surgical therapy associated with the use of laser therapy, as demonstrated by several studies, seems to improve and facilitate the healing of the treated sites [35]. Laser application is a relatively new modality that can be effectively used in addition to mechanical methods for the treatment of peri-implantitis. This is a therapeutic technique that uses laser energy to trigger a biochemical change in the cell membrane [36]. In dentistry, different types of lasers are used, characterized by different wavelengths that interact with different substrates, and therefore have different affinities with the various target tissues that make up the oral cavity. For example, neodymium YAG lasers (solid state), can be used, whose wavelength interacts with melanin and hemoglobin. The erbium YAG laser (solid state) demonstrates a greater affinity with water, and the carbon dioxide laser (CO2, gaseous state) uses water e hydroxyapatite. The diode laser interacts with the dark pigments of melanin and hemoglobin [37]. Laser therapy replaces the manual removal of tartar and plaque both from the surface of the teeth and from inside the gum pockets. Laser therapy involves the use of a photosensitizing substance when the laser passes. The function of this substance is to adhere to the membrane of bacteria. When the laser passes (diode laser, with a wavelength of 850 nanometers), the solution attached to the microorganisms is activated (photosensitizing reaction) and the colonies of residual bacteria are coloured blue. The laser beam then completes the procedure by destroying these colonies, thanks to the formation of active oxygen that breaks or dissolves the membrane of each bacterium.

The combination of manual curettage and antimicrobial photodynamic therapy performed with the laser allows the correct balance of the bacterial flora to be restored within the gum pockets. The use of lasers in periodontology is a relatively new system [38].

## 2. The D-BioTECH Technique

Adequate oral hygiene merged with timely therapeutic interventions avoid the progression of periodontitis, and may restore tooth health.

Good hygiene maintenance is the most demanding phase of non-surgical periodontal therapy, both for the patient and the operator. The risk to the health of periodontal and dental tissues is given by the ineffective management of bacterial biofilm, which often results in difficulty because of the presence of gum recessions, in addition to dentinal hypersensitivity. Discomfort in response to tactile, chemical, and physical stimuli makes proper domiciliary plaque control difficult.

It is therefore appropriate, during the therapeutic procedure, to alert the patient to the most effective technologies for the control of bacterial biofilm. The “Tailored Brushing Method”, as previously reported by Nardi et al. [39], explains how a personalized and predictable therapeutic approach helps to restore the patient’s periodontal health. In this study, carried out in 2016, all patients maintained a fairly satisfactory level of home oral hygiene. A greater reduction in the Bleeding on Probing Index was observed in the 100 patients enrolled in the test group (“Tailored Brushing Method”) compared to the 100 belonging to the control group; data were consistent with the points of the Detected Plaque Index. Data demonstrated the high efficacy of the personalized approach in terms of greater adherence to therapy, higher patient satisfaction, and the effective clinical improvement of oral hygiene levels [39].

Notably, the same biomedical devices may alter the normal biofilm and health of periodontal tissues; in fact, there are several impairments of quantitative and qualitative bacterial flora that can be iatrogenic, resulting in a significant increase in lactobacilli, staphylococci, and streptococci.

D-BioTECH, an acronym for Dental BIOfilm Detection Technique, is a Tailored Brushing Method that ensures a proper approach is conducted in patients with periodontal diseases. In this technique, the operator observes the variation of clinical signs by plaque detectors and other supports, sharing with the patient the topography of the bacterial biofilm, and discussing the best ways to remove it with proper methodologies.

D-BioTECH is applicable in non-surgical periodontal therapy: the aim of this treatment is to reduce local infections through the decontamination of the implant surface and the maintenance of good plaque control. However, the decontamination of implant surfaces is not particularly easy to achieve. In the scientific literature, many different approaches have been proposed to face dental pathologies and related gingival diseases. The D-BioTECH method is innovative because it involves aspects related to both the patient and to the dental team. Moreover, the D-BioTECH concept is based on minimally invasive dentistry; treating the given periodontal issue by means of air-polishing is the optimal choice as an alternative to the use of curettes, scalers, or polishing pastes. The D-BioTECH protocol also involves a fluorometric plaque detector to clearly check the dental surfaces that patients cannot reach for optimal plaque removal.

### Promising Approach to Oral Hygiene According to the D-BioTECH Concept

In recent years, there have been a number of studies on tailored approaches to teeth brushing and oral hygiene. Such studies were mainly carried out by researchers from the University La Sapienza of Rome. In 2017, the D-BioTECH approach was compared to the traditional methods of oral hygiene, and this interesting approach was applied in a case report of a 12-year-old boy with an orthodontic appliance. According to the D-BIOTECH clinical approach, patients should be followed by the same dental hygienist, and the bacterial biofilm should be detected by means of the Plac-o-Tect fluorescein plaque detector at baseline (T0) and follow-up (Tx). The clinical fluorometric results must be shown to the patient by the dental professionals so that the patient can self-evaluate their ability to perform domiciliary oral hygiene. The use of air-polishing offers a more accurate method of cleaning of such sites, leaving only traces of bacterial biofilm [40,41,42,43,44]. Nevertheless, different substances and techniques could be used to decontaminate the tooth’s surface, such as tetracycline paste, citric acid in different concentrations, chlorhexidine, hydrogen peroxide, sodium bicarbonate jets, ultrasound, and other effective methods. Recently, novel studies have been carried out to assess whether antimicrobial photodynamic therapy could effectively support conventional mechanical methods in the treatment of oral/gingival inflammatory condition. Typically, the subjects recruited for these studies were divided into two groups (test vs. control). The use of laser therapy and laser-aided decontamination involve the application of the D-BioTECH protocol, as patients need to be able to monitor their state of plaque control with a fluorescein plaque detector probe. To optimize such protocol, a better knowledge of the home protocol for managing oral health should be carefully disclosed to patients. The personalized approach reported by Nardi et al. aimed to describe the TBM (Tailored Brushing Method), a personalized and shared oral health maintenance protocol. This method involves the use of active oxygen-based toothpaste and continuous interactions with the patient. In the TBM, plaque indexes, bleeding with or without suppuration, and probing depth (PD) should be monitored at three different times. In a recent study, using TBM and D-BioTECH methods together, the results collected showed how the laser diode therapy with the addition of the D-BioTECH clinical approach had several advantages over the traditional methods, since the periodontal tissues presented more effective and faster healing compared to the tissues treated with traditional mechanical methods [44,45,46,47]. The TBM has also been linked to the innovative use of novel oral hygiene approaches, such as the use of ozonated olive oil mouthwash. This method, in fact, has been found to be more effective on salivary MMP-8 reduction than scaling and root plaining alone [48].

During the examination of the patient in the dental practice, great attention should be paid to the coexistence of pathologies related to the oral cavity. The omission of a dental examination or the possible elimination of odontogenic foci may affect the results of the general diagnostics and subsequent treatments. Measuring the levels of NO in the exhaled air seems to be a useful diagnostic method.

A patient undergoing several different dental treatments must be informed on the complexity of the therapy if it varies over time, and on the difficulties in maintaining home hygiene based on the type of orthodontic technique and device chosen. As an example, orthodontic treatments can use materials that modify bacterial microflora; therefore, it necessary to modulate the use of the domiciliary tools to manage oral hygiene according to the orthodontic stage and the ability of each patient to follow the domiciliary protocols. It is therefore advisable for a patient undergoing their first domiciliary treatments to follow the D-BioTECH approach, which means a “tailor-made personalized and shared” hygiene protocol. The D-BioTECH approach will allow the same patient to perform a careful self-evaluation of the quality of the oral hygiene practice. This self-evaluation involves understanding the care required to maintain dental interproximal spaces, local mineralization, the pigmentation of oral surfaces, and so on. It is not necessary to suggest specific and stereotyped brushing movements, but it is suggested that a shared choice is decided with the patient regarding the best technique and instruments to use on the basis of their specific clinical and anatomical dental conditions. In conclusion, patients should not hold a passive role; the therapeutic choices suggested by oral professionals should take the form of a discussion.

## 3. Conclusions

In September of 2016, the World Dental Federation (FDI) published an executive summary defining “oral health” as “multi-faceted and includes the ability to speak, smile, smell, taste, touch, chew, swallow and convey a range of emotions through facial expressions with confidence and without pain, discomfort and disease of the craniofacial complex”. In this landscape, the FDI also suggested that patients must be involved in the learning (e.g., chairside active demonstrations) of skills and methods to “personalize” their own approach to oral hygiene. In support of this thesis, several studies have been carried out; nevertheless, the team of authors of this article have greatly improved this concept, reporting the “TMB: Tailored Brushing Method”. This concept has been included and improved in our work, developing the D-BioTECH method. Undoubtedly, it could represent a turning point in the practice of oral hygiene, both for the patient and for the clinician. Visual-based communication is crucial to this new clinical approach. This means that the operator can plan and manage the intervention in a less invasive and more personalized way, and use the best-performing instrument or operating technique for the retention of bacterial biofilm.

However, it must be noted that the D-BioTECH method has some critical points. For example, the protocol cannot be standardized, as it is adapted to the needs of individual patients.
